# Academic performance depends on chronotype: Myth or reality?

**DOI:** 10.18231/j.ijcap.2019.103

**Published:** 2019

**Authors:** Indla Yogananda Reddy, Rajani Santhakumari Nagothu

**Affiliations:** 1 Dept. of Physiology, ESIC Medical College, Hyderabad, Telangana, India; 2 Dept. of Physiology, MedCiti Institute of Medical Sciences, Medchal, Telangana, India

**Keywords:** Academic Performance, Chronotype, Morningness and Eveningness, Questionnaire

## Abstract

**Introduction:**

Chronotype refers to an individual’s preference in the timing of sleep and wakefulness. Aim: This study targets to notice the relation between chronotype and academic performance of the first year MBBS students.

**Objectives:**

1. To ascertain the chronotype of first year MBBS students 2. To harmonize the chronotype and internal assessment theory marks. This is an educational observational study, conducted on hundred first MBBS students in a medical college, located at sub-urban area of Hyderabad. Self-assessment

Morningness and Eveningness Questionnaire (MEQ) developed by James A. Horne and Olov Ö stberg is applied in segregating the students into different chrontype groups. MEQ is having 19 multiple choice questions with four to five options against each question and with specific score for each option and the scores can vary from zero to six. After completion of MEQ test, students were grouped accordingly. Mean of the internal theory assessment marks and percentage of theory attendance for each chronotype group was calculated. The relation between the chronotype, theory marks and percentage of theory attendance was ascertained.

**Results:**

There is no correlation between the MEQ scores and the first internal assessment theory marks, though the marks are positively correlated with the theory attendance percentage.

**Conclusion:**

Academic performance depends on chronotype, it is a myth not real.

## 1. Introduction

In Indian mythology, Brahmamuhurtha i.e. the time of creator is one and half hour before the sunrise, is considered as auspicious time for doing any practice like studying, initiating a new project etc. It is a general opinion existing in the society that early to bed and early wakeup will help in putting better performance in professional life and this can be attributed to the creator’s time. Human beings are diurnal i.e. day timers because of the endogenous biological clock. Chronotype is phonotypical behavioral manifestation of endogenous timing system i.e. circadian clock. Individual variation within our biological clock drives our morning or evening preferences, thereby making us into ‘morning larks’ or ‘night owls’. Chronotype has a genetic basis 1–4 that can be altered by the external cues like; physical growth, gender, environmental factors, and the physical activity. Chronotype is different in men and women and it keeps on changing with advancing in age in the same individual.

## 2. Aim

This study targets to notice the relation between chronotype and academic performance of the first year MBBS students.

## 3. Objectives

1. To ascertain the chronotype of first year MBBS students.
2. To hormonise the chronotype, internal assessment theory marks and theory attendance percentage.

### 3.1. Material

### 3.2. Study design

Questionnaire based Educational Observational Study.

### 3.3. Duration

3 months.

## 4. Sample size/Study group

100 1*st* year MBBS students.

Institutional Ethical Committee approved the study.

### 4.1. Exclusion criteria

1. Students who were absent on the day of administration of the test.
2. Who appeared for the theory exam but absent on the day of chronotype test or vice versa.
3. Who did not complete the chronotype test properly like encircling two answers for one question/left one or more questions unanswered.

## 5. Materials and Methods

Students were recruited in the present study after obtaining the written informed consent. The self-assessment morn- ingness and eveningness questionnaire (MEQ) implemented in the present study was developed by James A. Horne and Olov Ostberg.^10^ Institutional prevalidation was carried out before the administration of MEQ in the present study. MEQ is having 19 multiple choice questions with four to five options against each question and with specific score for each option and these scores are from zero to six. Students should mark only one suitable option for each question. After completion of MEQ test, students were grouped under different chronotypes based on their total MEQ scores. Mean of the internal theory assessment marks and theory attendance percentage for each group was calculated. Finally, the relation between the chronotype, theory marks and percentage of theory attendance was ascertained.

## 6. Results

Eleven students were absent on the day of conduction of MEQ test. Based on the total scores, all the 89 students were grouped under: 2 as definite evening, 11 as moderate evening, 62 as intermediate and 14 as moderate morning type. None of them were in definite morning group. Three students did not appear for the physiology internal theory assessment examination & out of these three, one was absent even for the MEQ test. Finally, 87 students were took part both in the physiology internal theory assessment examination and the MEQ test. The results were analyzed by using Statistical Package for the Social Sciences (SPSS).

## 7. Discussion

**Graph 1:**
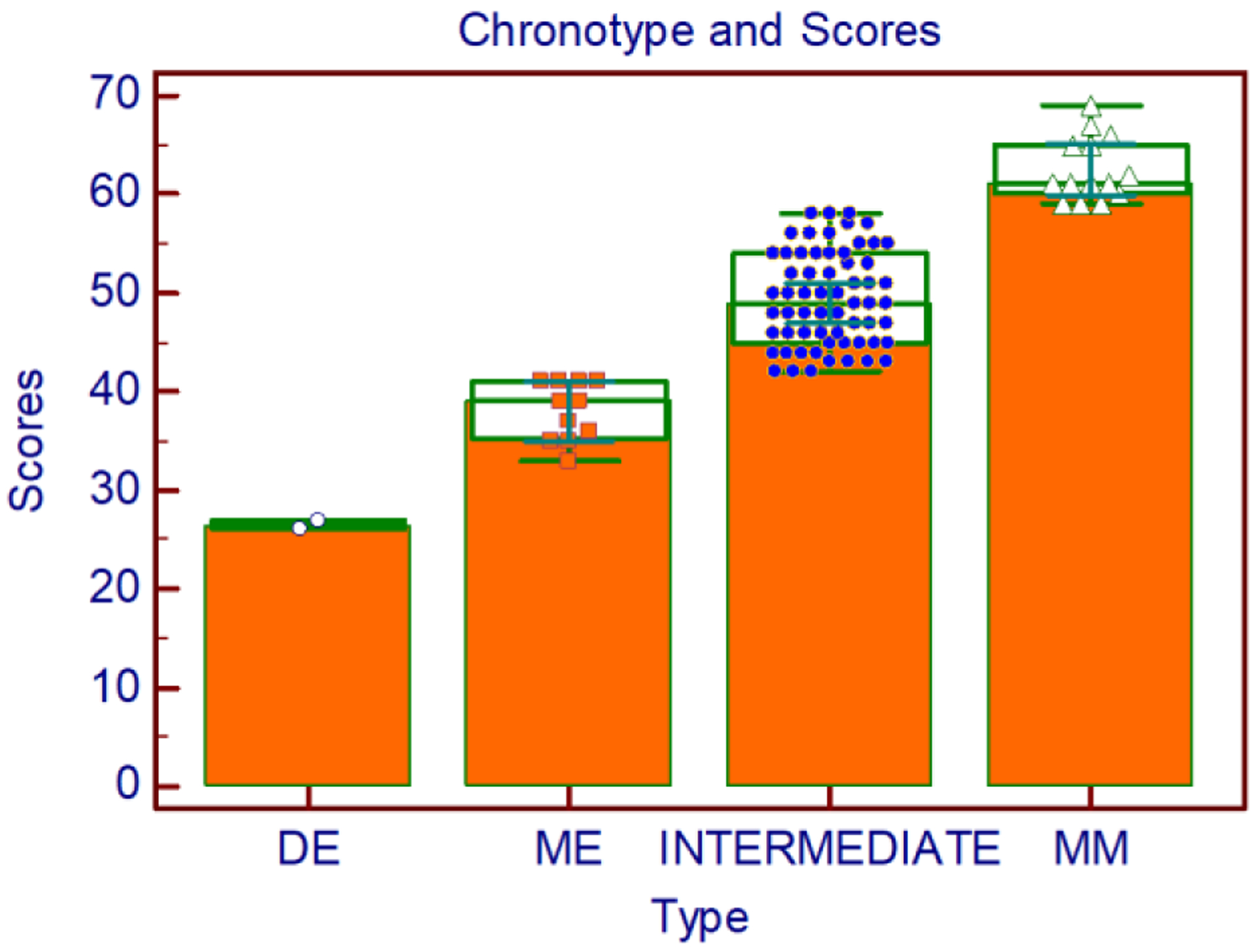
Mean & standard deviation of MEQ scores of different chronotype groups

First, based on the total scores obtained in MEQ, as per the Table 1, students are divided into four chronotypes. Not even a single student was in definite morning type, and only 2 are under definite evening type as shown in table 2. Second, gathered the internal theory assessment marks, theory attendance percentage of all the students who have participated in the study and calculated the mean of the same for each chronotype group separately. Third, we perceived that there is no interdependency between internal assessment theory marks and the chronotype of the students ( Graph 1), and these findings are in line with the earlier research reported. 11 Unlike practical/clinical assessment the theoretical assessment is based on crystallised intelligence. Chronotype influences the cognitive performance especially in actions that require the fluid intelligence than on those using crystallised intelligence. 12–16

Crystallised intelligence is based on education, and the theoretical academic performance is part of education and hence chronotype is not effecting the performance of the students in their theory exams. On the contrary to our findings evening type students have scored low grades, 17–20 high grades as well. Under accomplishment can be attributed to the mediocre attention/orientation, poor quality of sleep, low attendance, uncomfortable timings of classes & examination. 21,22 That is because the theory classes and the exams are generally conducted during the morning hours when the evening type are still not at their peak state of attention/concentration. Which was not evident in the present study. Geneticalley for schizophrenia, educational achievements, body mass index and chronotype is one and the same. 20 This should trigger our thoughts on, to trace the connection between, the usually intelligent schizophrenics and their chronotype.

**Graph 2:**
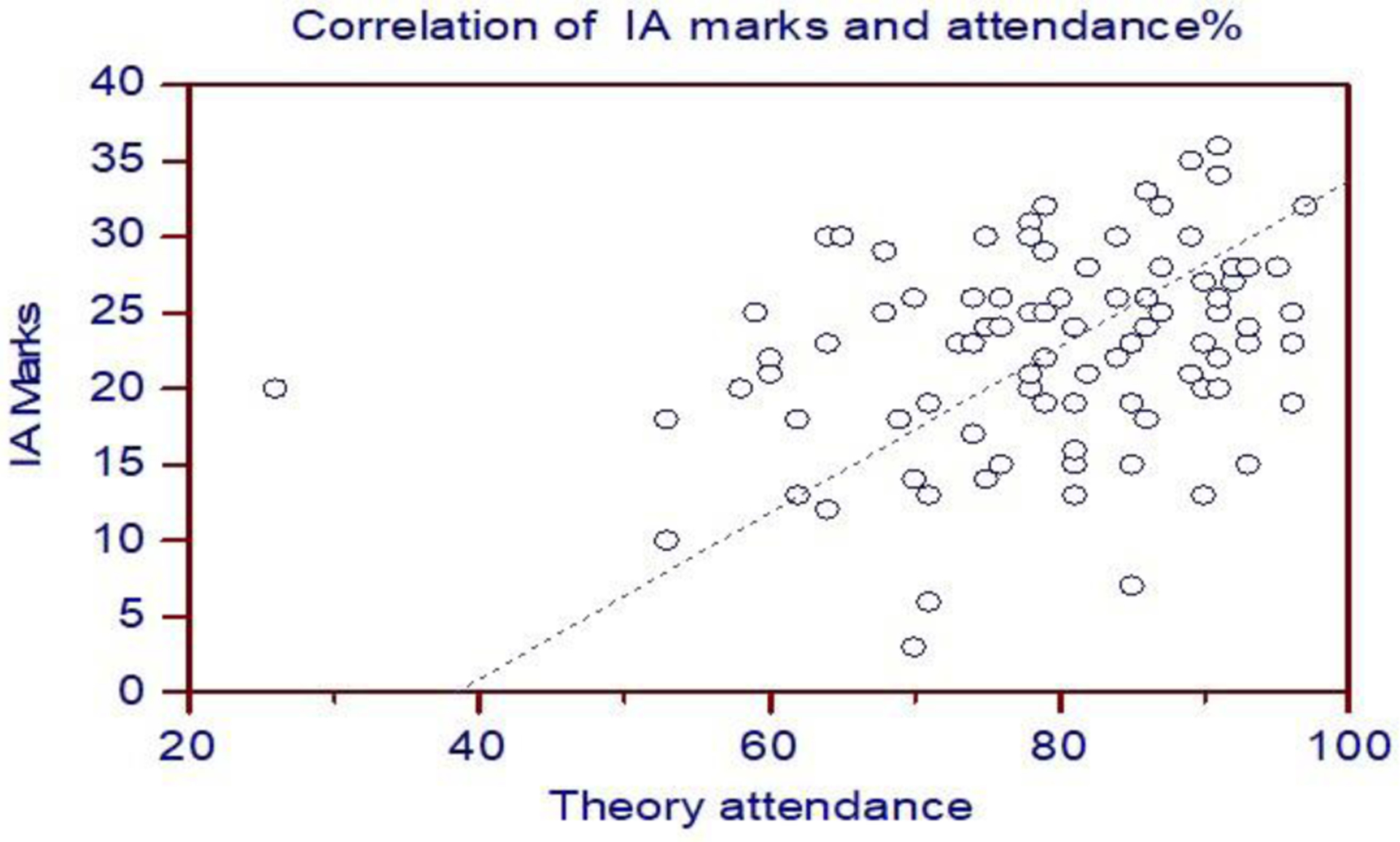
Relation between theory attendance percentage and the internal assessment marks Note: Correlation Coefficient (r) is 0.27 and P=0.0067, so there is association between Internal Assessment Marks and the Theory Attendance Percent- age.

There are ascertained evidences on the better per- formance by the morning type. 23–25 The better accom- plishment by the morning type can be attributed to, sufficient sleeping hours, better quality of sleep, and the fair attention during the class and the examination hours. Genetic component linking the morning type and better academic performance to be pinpointed. The number of study hours and study time per day will also effect their performance apart from various other factors. Graph 2, shows that there is, a positive association between the percentage of attendance and the theory marks individually for each student. The students who secured higher marks are also with higher attendance. These students are representing different chronotype groups, so we cannot attribute chronotype to their marks. Not a single chronotype group has significant high theory marks or attendance percentage over the other groups. Based on the results of the present study and the references mentioned above we can clearly elucidate that good, normal and poor performers are present in all variants of chronotypes. So it is difficult to justify that, the academic performance depends on chronotype of the students, there are other factors as well that can influence the academic achievements. The present study will definitely act as a base in bringing out the seminal works on chronotype and performance.

From the time immemorial, the Indian education system is teacher (Guru) centered, where students/disciples are supposed to attend the classes as per the schedule of their guru. This teacher centered traditional teaching is now at stake because the students have got their own priorities for study hours, attending the classes, sleeping time etc. Not realizing this, we as parents/teachers are instructing our children to get up early in the morning & study and to go to bed early in the evening to which the endogenous biological system in some children is completely unacclimatised. This kind of student’s unfriendly schedule is the prime cause for the psychological breakdown and low grades for many students. Sometimes it is the prime culprit for the students to committee suicide because of the mismatch between the inherent biological clock and the imposing schedule of the college or school. Some of the recent past incidents are very much evident for the same. Now it’s high time for the policy makers to amend the existing teacher comfort time table to the student’s friendly time table as per the requirements, needs and comforts of the students. We as a teacher/parent should also give respect to the children’s priorities. Education is not all about grades and ranks but ethics, values and if we ourselves don’t follow them by respecting the kids needs then how can we expect them, to be benefactors of the humanity when they grow up.

## 8. Conclusion

Academic performance is independent of chronotype.

## 9. Study Limitations

Scientifically if each chronotype group has at least 30 students as per the research norms, then the results would have had more authentication. We did not inquire about the number of study hours, time of study hours, and amount/quality/time of sleep.

## Figures and Tables

**Table 1:** Classification of Chronotypes based on MEQ scores

S. No	Total score	Chronotype
1	16–30	Definite E vening type
2	31–41	Moderate E vening type
3	42–58	Intermediate type
4	59–69	Moderate M orning type
5	70–86	Definite M orning type
6	*≤* 41	Evening type
7	*≥* 59	Morning type

**Table 2:** Mean & SD for MEQ scores, IA marks & attendance percentage for each chronotype group

Chronotype	No. Students	MEQ Scores	IA-Marks	Attendance %	Significance
Definite-Evening	2	26.50*±*0.70	22.50 *±*4.94	79.5 *±*0.70	NS
Moderate-Evening	11	38.0*±*2.93	20.45 *±*5.29	78.08 *±*12.73	NS
Intermediate	62	49.46*±*4.70	22.50 *±*6.49	79.18 *±*12.23	NS
Moderate-Morning	14	62.50*±*3.27	25.38 *±*7.74	79.28 *±*14.62	NS
